# Nickel nanoparticle-decorated reduced graphene oxide/WO_3_ nanocomposite – a promising candidate for gas sensing

**DOI:** 10.3762/bjnano.12.28

**Published:** 2021-04-15

**Authors:** Ilka Simon, Alexandr Savitsky, Rolf Mülhaupt, Vladimir Pankov, Christoph Janiak

**Affiliations:** 1 Institut für Anorganische Chemie und Strukturchemie, Heinrich-Heine-Universität Düsseldorf, 40204 Düsseldorf, Germany; 2 Chemical Faculty, Belarusian State University, Leningradskaya str. 14, 220050 Minsk, Belarus; 3 Freiburg Materials Research Center and Institute for Macromolecular Chemistry, Albert-Ludwigs-University Freiburg, 79104 Freiburg, Germany

**Keywords:** gas sensing, magnetic measurements, nickel nanoparticles, reduced graphene oxide, tungsten oxide

## Abstract

We report for the first time the combination of WO_3_ sensing elements with a non-noble metal–carbon composite, namely a nickel metal nanoparticle–carbon composite (Ni@rGO). Previous work with WO_3_ had used either NiO (as part of the WO_3_ lattice), solely carbon, Pd-surface decorated WO_3_ (Pd@WO_3_), or Pd or Pt@carbon@WO_3_. We demonstrate the gas response for pure WO_3_, rGO/WO_3_ and Ni@rGO/WO_3_ sensing elements towards NO_2_ and acetone in air as well as towards CO in N_2_. The addition of 0.35 wt % Ni@rGO composite to WO_3_ enables the increase of the sensory response by more than 1.6 times for NO_2_ vapors. The gas response towards acetone using 0.35 wt % Ni@rGO/WO_3_ composite was 1.5 times greater for 3500 ppm than for 35,000 ppm acetone. For 0.35 wt % Ni@rGO/WO_3_ composite and CO gas, a response time (*T*_res_) of 7 min and a recovery time (*T*_rec_) of 2 min was determined.

## Introduction

Toxic gases as well as volatile organic compounds (VOC) are known air pollutants and their emissions are harmful for humans and ecosystems [1]. Sensor materials that can detect the type and concentration of these gases are therefore needed in various kinds of environments and industries [2]. A gas sensor should be highly sensitive and highly selective with a fast response and recovery rate. Also, it should work at low cost and with low power consumption [3]. In comparison to conventional gas sensors, nanostructure-based gas sensors are more sensitive because of their increased detection area [4]. The most common mode used in gas sensing is the resistance mode, where the change in sensor resistance during exposure to the gas is measured directly [5]. Gases can either be oxidizing, such as NO, N_2_O, NO_2_, O_3_, and Cl_2_, reducing, such as H_2_S, NH_3_, CO, H_2_, SO_2_, and CH_4_, or rather inert, such as CO_2_ [6–7]. VOCs are organic molecules such as acetone, ethanol, and formaldehyde [8–9].

Metal oxide semiconductors (MOS) are the most commonly used gas sensors [10]. MOS can be divided into n-type and p-type MOS. In n-type MOS electrons are the majority charge carriers, while in p-type MOS holes are the majority charge carriers [6]. The exposure to reducing gases causes a decrease of resistance in n-type MOS and an increase of resistance in p-type MOS and vice versa [8]. MOS have certain advantages such as fast response time and excellent sensitivity towards all kinds of gases [11]. The major disadvantages of MOS are their poor selectivity and high operating temperatures of 200 to 400 °C, which means a high power consumption [4]. WO_3_ is a wide-bandgap [12–13] n-type semiconductor [14–15] with good sensitivity towards NO_2_ [16] and CO [17].

Known successful routes to improve the MOS gas sensing performance are doping with transition metals, decoration with noble metals, formation of heterojunctions, or size reduction [18–19]. Doping of WO_3_ with nickel improves the humidity sensing compared to neat WO_3_. Attributed to a greater number of electrons donated by Ni atoms, higher surface area, and smaller bandgap energy, Ni-doped WO_3_ has a faster response, higher sensitivity, and greater stability than pure WO_3_ [20]. WO_3_ decorated with palladium nanoparticles on the surface can be used as an improved and reusable gas sensor for NH_3_ [21].

Metal oxide semiconductor junctions can either be formed between two p-type MOS or two n-type MOS (p–p/n–n homojunctions) or between a p-type MOS and an n-type MOS (p–n heterojunctions) [6,18]. The p-type MOS NiO is not a very popular gas sensing material, because p-type MOS have, in general, a lower gas response than n-type MOS, such as WO_3_, ZnO, or SnO_2_ [22–23]. But p-type MOS are ideal doping agents [24]. NiO combined with WO_3_ forms a p–n heterojunction, which improves the gas sensing abilities significantly [25].

Carbon-based materials are also promising gas sensors, because of their high surface area and high chemical and thermal stability [26–27]. Pristine graphene is a good conductor but rather inactive for gas sorption, because it has only a few functional groups on its surface, which limits the chemisorption of gas molecules [28]. Graphene oxide (graphite oxide, GO), in contrast, has numerous oxygen functionalities and few remaining π bonds and is therefore electrically insulating [29]. GO can be reduced (reduced graphene oxide, rGO) chemically or thermally. Through the partial removal of oxygen groups, the conductivity can be restored. Additionally, defects and vacancies are created [26]. Because of the ultra-high surface area per atom and the high electron transport along the graphene plane, rGO has a rapid and high response to gas molecules at room temperature [30]. A disadvantage of rGO gas sensors is the long recovery time because of the high binding force between gas molecules and the graphene material [31]. rGO is a p-type semiconductor and can be used for gas sensing of low concentrations of NO_2_ at room temperature [32].

The combination of MOS with graphene materials can improve the gas sensing abilities [33–34]. MOS prevent graphene from agglomerating, which leads to a higher specific surface area. Graphene can control the size and morphology of MOS during the synthesis and decreases the resistance of MOS, which leads to a rapid electron transfer from the surface reaction of the target gas with the MOS to the electrodes [35]. Additionally, MOS and graphene can form junctions at their interface. For example, p–p homojunctions can be formed between NiO and rGO to increase the gas sensing responsivity and sensitivity towards NO_2_ [36]. In the combination of WO_3_ and rGO, p–n heterojunctions are formed. This leads to an increased NO_2_ response at room temperature [37]. Overall, MOS@rGO gas sensors are more selective and sensitive with a faster response and recovery rate even at room temperature [8].

The sensing performance of MOS@rGO can further be improved by either chemical doping or by combination with a transition metal as ternary component [38]. Iron oxide-doped WO_3_ films showed improved NO_2_ sensing at room temperature, when adding a layer of 16 nm p-type rGO on the metal oxide film [39]. Nickel-doped SnO_2_ nanoparticles loaded with graphene have an enhanced acetone response at 350 °C with increased graphene loading level (best at 5 wt % graphene) [40]. ZnO nanostructures doped with nickel and rGO were used for hydrogen sensing at 100 °C [34].

The decoration of MOS with a noble metal, such as Pd or Pt, improves the sensitivity, response time and working temperature of MOS/rGO systems [15,41]. TiO_2_/rGO decorated with Pd and Pt nanoparticles was successfully used in the gas sensing of hydrogen gas [19]. The decoration of WO_3_/rGO nanosheets with Pt nanoparticles yielded a faster response for acetone at 200 °C [42]. With the addition of Ag nanoparticles to a dispersion of SnO_2_/rGO, the working temperature was dropped from 55 °C to room temperature in the gas sensing of NO_2_ [43]. (For further examples and comparison with other gas sensors see Table S1 in Supporting Information File 1.)

The ternary Ni@rGO/WO_3_ nanocomposite was synthesized and tested in comparison to pure WO_3_ and rGO@WO_3_ regarding the response to the oxidizing gas NO_2_ (10 ppm in air) and the VOC acetone (35,000 ppm in air). Gas response to CO and recovery times were also determined. An examination of the influence of different gas concentrations on the gas response were measured for 3500 ppm and 35,000 ppm acetone.

## Results and Discussion

### Ni@rGO synthesis

The synthesis of nickel nanoparticles is well known and different methods such as thermal decomposition [44] or reductive hydrogenation [45] are used. Nickel nanoparticles with sizes below 10 nm can be easily synthesized from the precursor material bis(1,5-cyclooctadiene)nickel(0) (Ni(COD)_2_) in different ionic liquids without any additional stabilizing or reducing agents [46]. Ionic liquids have the ability to exfoliate graphene oxide into single sheets. Thus, a higher surface area can be achieved [47]. Thermally reduced graphene oxide was tested before with different metals in ionic liquids [48–49]. The decoration of nanoparticles on rGO can be achieved in situ or by mixing previously prepared solutions [50].

**Figure 1:**
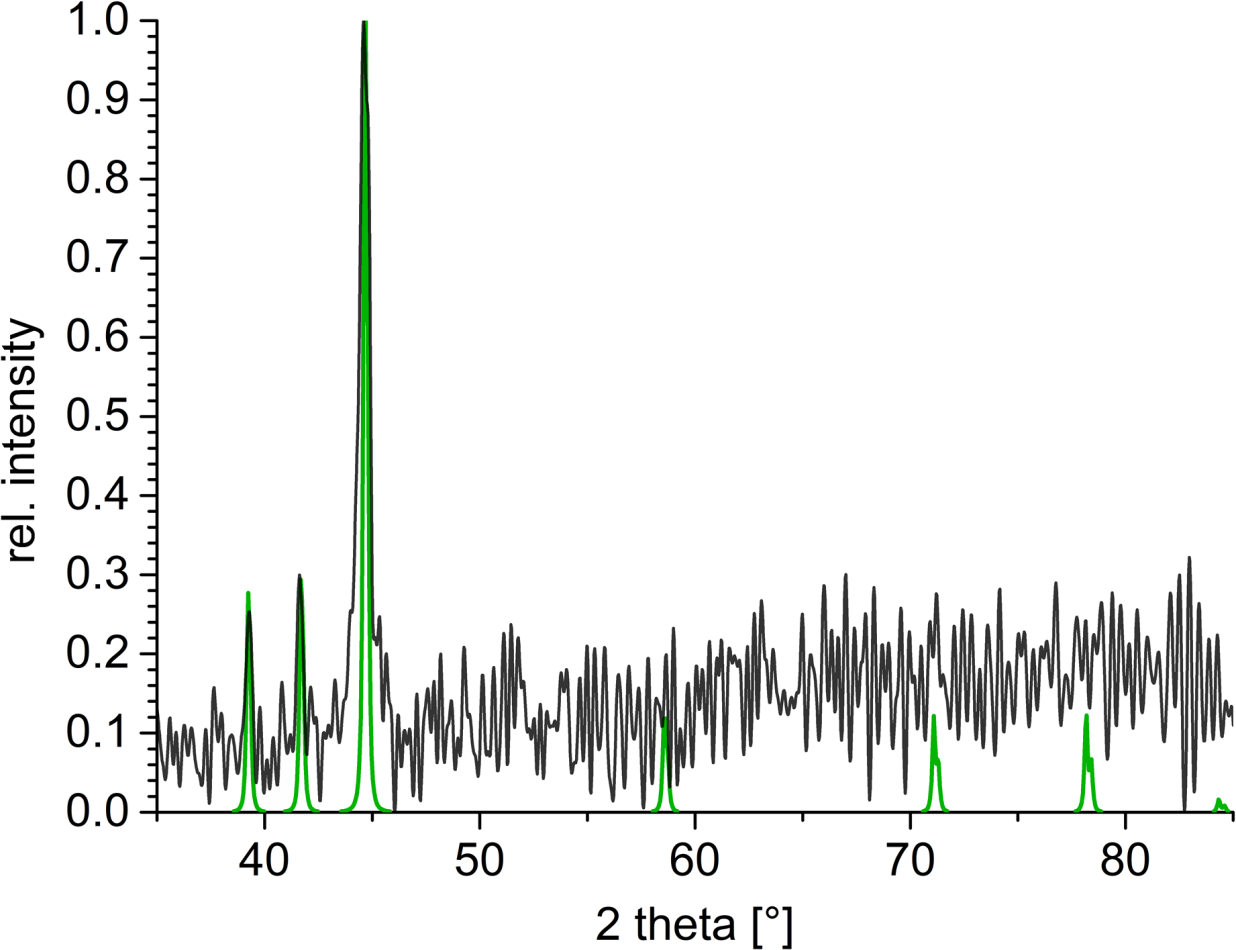
P-XRD pattern of Ni@rGO obtained from a 0.5 wt % dispersion of Ni(COD)_2_ in [BMIm][NTf_2_] (space group of nickel: *P*6_3_/*mmc*).

Here, we chose the ionic liquid [BMIm][NTf_2_] for an in situ microwave decomposition approach with rGO synthesized from reduced graphite oxide at 400 °C. It is extremely important that the used rGO is thoroughly dried because of the oxyphilic nature of nickel nanoparticles. Therefore, before the nanoparticle synthesis, the rGO was dried using a turbo molecular pump at 5 × 10^−7^ mbar for several days. Then rGO was dispersed with Ni(COD)_2_ in [BMIm][NTf_2_] to gain 0.5 wt % metal nanoparticles and 0.5 wt % rGO. In order to stir the reaction mixture during the microwave decomposition, 0.5 wt % rGO could not be exceeded. The obtained nanomaterial was analyzed using powder X-ray diffraction (P-XRD). The P-XRD pattern shows the reflexes for hexagonal nickel (Figure 1).

**Figure 2:**
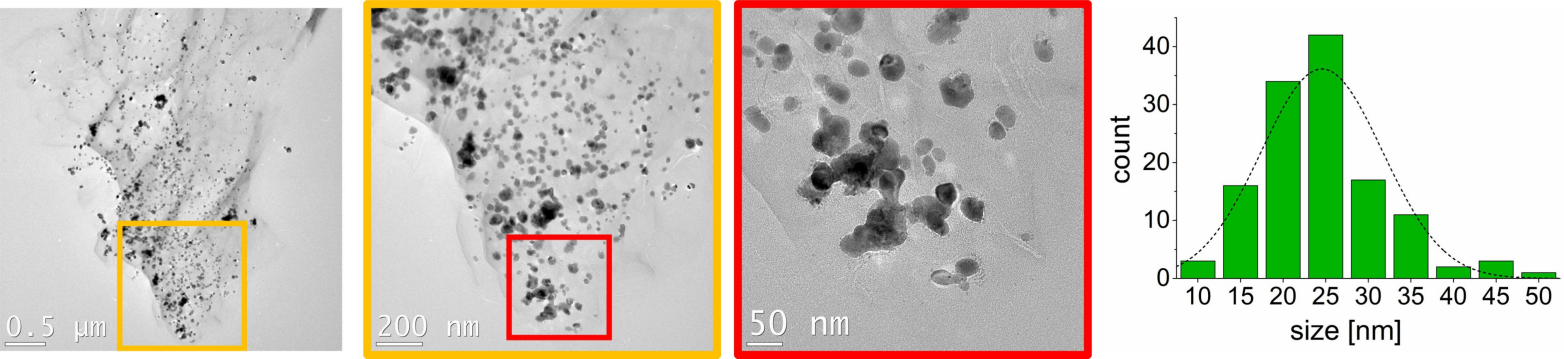
TEM images of Ni@rGO obtained from a 0.5 wt % dispersion of Ni(COD)_2_ in [BMIm][NTf_2_]. Particle size 25 ± 5 nm.

TEM images show spherical nickel nanoparticles, which are supported on top of rGO (Figure 2). The particles have a size of 25 ± 5 nm. All nanoparticles are supported on rGO. The particle size of Ni@rGO increased in comparison to pure nickel nanoparticles from [BMIm][NTf_2_] (size pure nickel nanoparticles 11 ± 2 nm) [46]. Nickel nanoparticles supported on pristine graphene sheets were synthesized with a size 35 ± 5 nm [51].

The nickel content was measured using atomic absorption spectroscopy (AAS). Ni@rGO contained 8% nickel. A metal loading between 5% and 20% on graphene oxide is common [49].

### WO_3_ nanopowder synthesis

**Figure 3:**
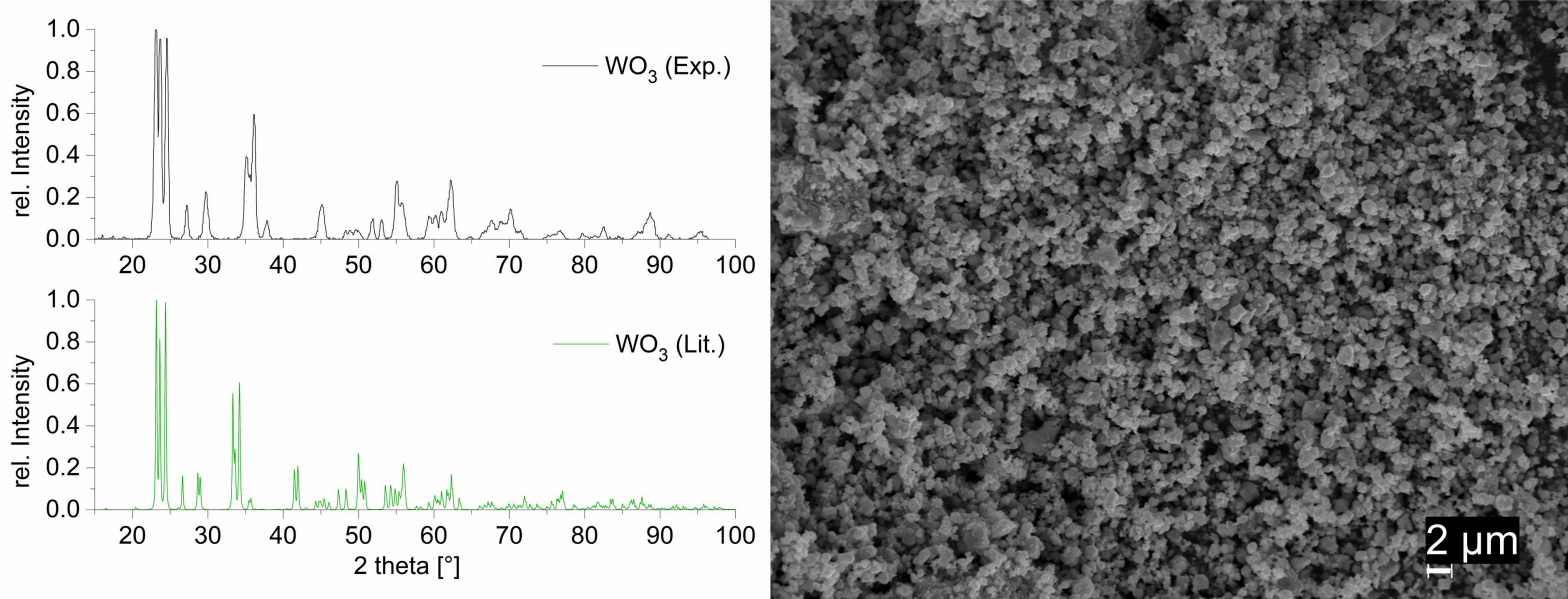
Left: P-XRD pattern of WO_3_ powder calcined at 600 °C for 2 h. (space group of WO_3_: *P*2_1_/*n*). Right: SEM images of WO_3_ powder calcined at 600 °C for 2 h.

The tungsten oxide nanopowder was prepared by a sol–gel method according to [52]. The phase composition was analyzed using P-XRD. The P-XRD pattern shows reflexes only of monoclinic tungsten oxide (Figure 3). Therefore, the thermal decomposition of the WO_3_ xerogel leads to the formation of only one crystalline WO_3_ phase without crystalline by-products. The average size of WO_3_ nanoparticle crystallites, calculated from the powder pattern using the Scherrer equation, is 40 nm. The SEM images show grains of WO_3_ nanocrystals in different sizes (hundreds of nanometers to several micrometers). Smaller grains are uniformly distributed on the surface of larger grains (Figure 3).

### Gas sensing measurements on gas permeable pellets

Ni@rGO was then mixed with WO_3_ xerogel and pressed into pellets to be tested in gas sensing measurements. Dry air was used as a reference gas. The electrical resistance was measured for the testing gas mixture and air. The response of a semiconductor sensor is the ratio between the electrical resistance in air and that in a gas medium. In the presence of reducing gases (e.g., acetone or CO), the sensor resistance decreases. In the presence of oxidizing gases (e.g., NO_2_), the electrical resistance increases [7].

**Figure 4:**
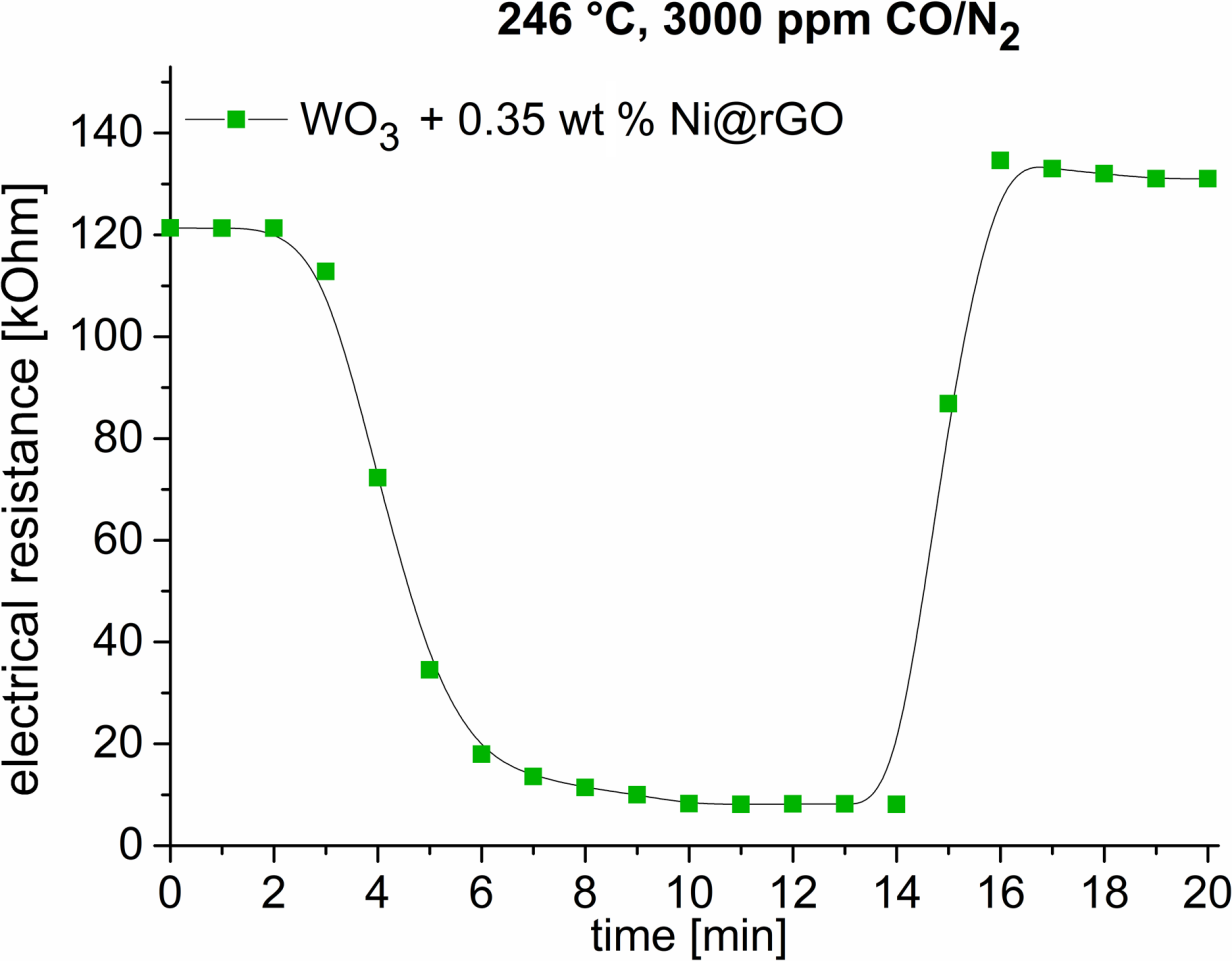
Time dependence of the sensor resistance values of the 0.35 wt % Ni@rGO/WO_3_ sample under exposure to 3000 ppm CO in nitrogen. Response time: ca. 7 min, recovery time: ca. 2 min.

Figure 4 shows the sensor characteristics for the Ni@rGO/WO_3_ composite in 3000 ppm CO/N_2_ at 246 °С. A high sensor response of *R*_air_/*R*_gas_ = 14.8 was detected (Figure S1 in Supporting Information File 1). It was found that a constant baseline resistance was observed before and after exposure. The response time (*T*_res_) is 7 min. The recovery time (*T*_rec_) is 2 min.

**Figure 5:**
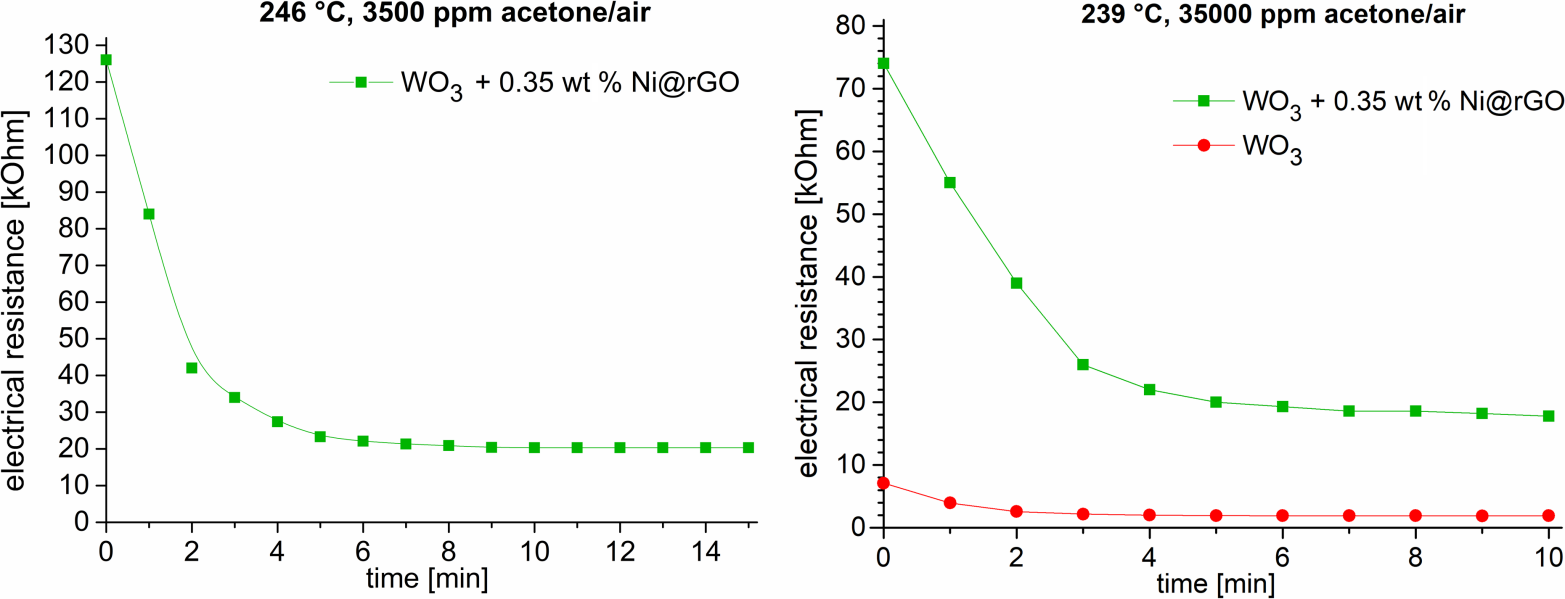
Left: time dependence of the sensor resistance values of the 0.35 wt % Ni@rGO/WO_3_ samples (green) under exposure to a mixture of 3500 ppm acetone vapor in air. Right: time dependence of the sensor resistance values of the 0.35 wt % Ni@rGO/WO_3_ (green) and WO_3_ (red) samples under exposure to a mixture of 35,000 ppm acetone vapor in air.

A sensor response of the Ni@rGO/WO_3_ composite of *R*_air_/*R*_gas_ = 6.20 in 3500 ppm acetone was detected (Figure 5 right, Figure S2 in Supporting Information File 1). For a higher acetone concentration of 35,000 ppm, the sensor response was lower with *R*_air_/*R*_gas_ = 4.2 (Figure 5 left, Figure S2 in Supporting Information File 1).

**Figure 6:**
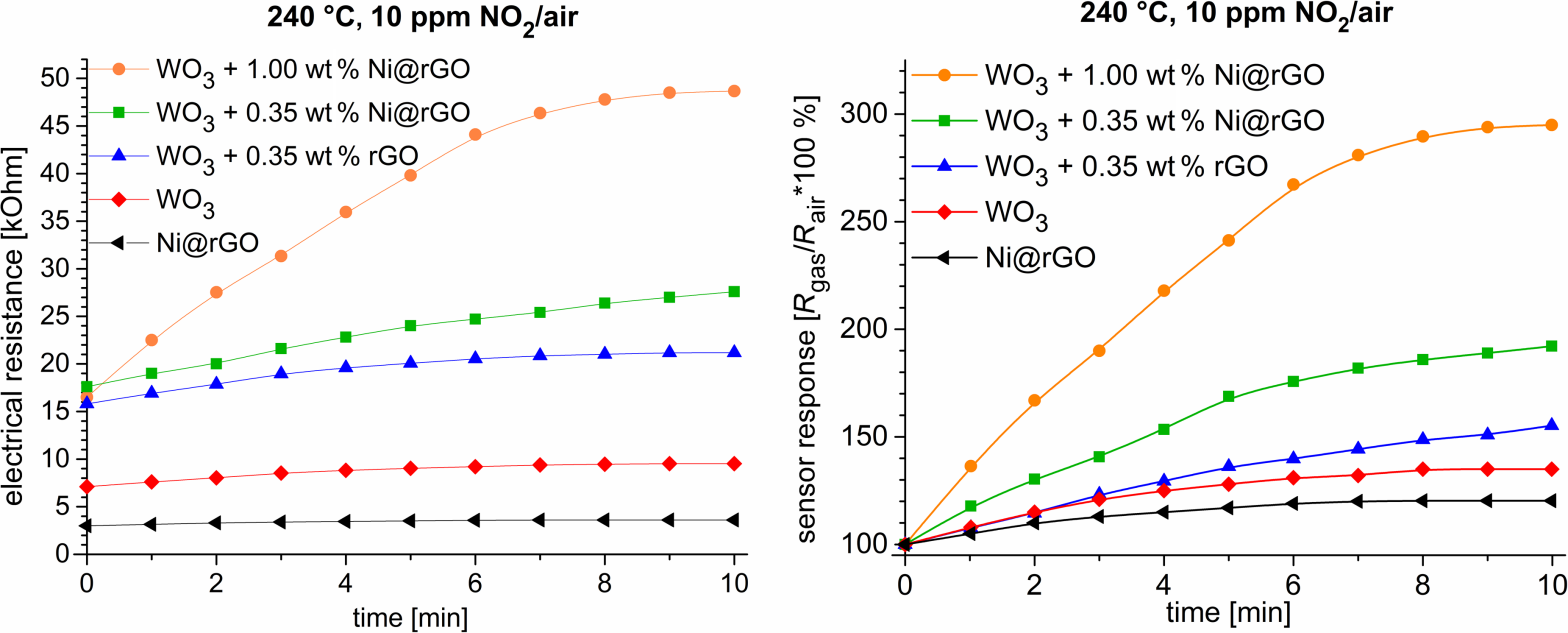
Time dependence of the sensor resistance values (left) and sensor response (right) of 1.00 wt % Ni@rGO + WO_3_ (orange), 0.35 wt % Ni@rGO + WO_3_ (green), 0.35 wt % rGO + WO_3_ (blue), WO_3_ (red), and Ni@rGO (black) samples under exposure to a gas mixture containing 10 ppm NO_2_ in air.

At 240 °C the electrical resistance of the 0.35 wt % Ni@rGO/WO_3_ sample in a gas–air environment containing 10 ppm NO_2_ increased 1.6-fold (from 17.6 to 27.6 kΩ, Figure 6 left).

Figure 6 (right) shows a sensor response curve for a sample that is a composite layer of Ni@rGO in the conductive polymer poly(3,4-ethylenedioxythiophene):poly(styrenesulfonate) (PEDOT:PSS). This layer is applied to a corundum substrate. In the case of NO_2_, the sensor response (*R*_gas_/*R*_air_) is, respectively, 2.9 for 1.00 wt % Ni@rGO + WO_3_, 1.9 for 0.35 wt % Ni@rGO + WO_3_, 1.6 for 0.35 wt % rGO + WO_3_, 1.4 for WO_3_, and 1.2 for Ni@rGO. Thus, the addition of Ni@rGO to WO_3_ enables the increase of the sensor response to NO_2_ and acetone vapor.

In contrast to sensors in which the sensing element consists only of rGO, sensors based on semiconductor oxide compositions, for example, WO_3_ with rGO, have a higher response. In the case of pure rGO, the restoration of the original parameters of the sensors may not be observed at all [8]. In addition, oxide-based composites are mechanically more durable and manufacturing sensors based on them seems to be more economically feasible due to the low content of graphene in the sensing element (up to several percent).

At present, there is no generally accepted mechanism of gas sensitivity of semiconductor oxide compositions with graphene. The reasons for the increase in the response and decrease in the operating temperature of metal oxides combined with non-oxidized graphene are synergetic effects between graphene and metal oxides as a result of chemical bonds between graphene and metal oxide. In the case of reduced graphene oxide (semiconductor), various reasons are considered, such as the appearance of p–n junctions that shift the Fermi level of the oxide. There is evidence of effective charge transfer between graphene and nanospheres through chemical bonds. Emergence of conducting channels from graphene layers is also pointed out, which increase the efficiency of charge carrier transfer in composites [8].

Based on the known literature data and the results obtained, it is possible to provide potential reasons for an enhancement of the sensitivity in the case under consideration. When Ni@rGO/WO_3_ sensors are exposed to NO_2_, with NO_2_, which is a strong oxidizing gas accepts electrons from WO_3_. As a result, NO_2_ transforms into NO_2_^−^ on the surface of WO_3_. This process leads to accumulation of holes and an increase in sensor sensitivity. In addition, the rGO/WO_3_ contact also plays an important role in charge transfer processes and leads to the enhancement in the gas sensing performance due to the synergistic effect between rGO (p-type) WO_3_ (n-type) [29]. In [37,53], the formation of C–O–W bonds at the interphase boundaries was observed, when studying a rGO/WO_3_ composite using XPS and Raman spectroscopy. Such bonds can play an important role also in our case with regard to charge transfer. It has been established that adsorption of NO_2_ molecules will cause upward band bending by capturing free electrons from the conduction band and shift the Fermi level of WO_3_ away from the conduction band toward the valence band. Processes on the WO_3_ surface make it possible for the work function of WO_3_ to lower to a point close to that of rGO [37]. The latter facilitates transfer of electrons at the rGO/WO_3_ interface. The continuous capture of electrons by chemisorption of NO_2_ gases at the surface of WO_3_ facilitates the charge transfer from rGO to WO_3_. At the expense of C–O–W bonds, hole transfer from WO_3_ to rGO will occur. Thus, it has been shown that C–O–W bonds are responsible for enhancing the charge carrier transfer rate [54]. In addition, graphene sheets in the composite create a hierarchical nanostructure and facilitate the diffusion of NO_2_ molecules, increasing the contacts and enhancing the chemisorption of the gas. rGO has large specific area and more active sites.

Gas sensors of n-type semiconductors based on oxides exhibit resistance changes induced by chemisorption of oxygen adions (O^−^ and O_2_^−^) that interact with reducing gases [55–56] such as acetone. A free electron appears in the conduction band of the semiconductor after the interaction of the chemisorbed oxygen adions and the target gas [57]. When acetone adsorbs on the surface of such a sensor material, preadsorbed oxygen adions are released according to the reaction [58]:


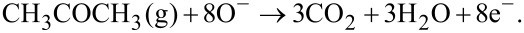


The same is true for CO [57]:


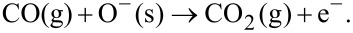


As a result, electrons that were trapped in the oxygen adions return back to the conduction band of WO_3_. Thus, the resistance of WO_3_ decreases upon exposure to these gases. An increase in the sensitivity to acetone of the entire composition 0.35 wt % Ni@rGO/WO_3_ may be associated with further electronic interaction between WO_3_ particles (n-type) and Ni@rGO (p-type). In this case, the transfer of electrons from WO_3_ to rGO leads to the formation of spatially separated regions of positive and negative charges (possibly, a p–n junction is formed). It was shown [59] that electrons that were transferred from the n-type semiconductor and stored in the rGO sheets are withdrawn upon exposure to gas, thereby restoring the hole concentration and p-type conductivity of rGO. This is made possible by the energy band alignment between the semiconductor and rGO, the electron acceptor functionalization of the analyzed gas, and the p-type conductivity of the rGO. Thus, the electron-depleted WO_3_ surface is more sensitive to the adsorption of acetone molecules and the transition of electrons from the adsorbed gas to the WO_3_ conduction band.

When considering the possible mechanism of the influence of nickel on the sensory properties of the Ni/rGO/WO_3_ composite, in addition to selective catalytic activity, it is also possible to refer to the existing explanation for the interaction of clusters of metal particles with a semiconductor. In accordance with the literature data, the role of nickel in increasing the sensory sensitivity is most likely associated with the spillover effect [60–61]. When interacting with a gas atom, the barrier height at the Ni/rGO interface decreases due to this effect (the possibility of electron transfer from nickel particles to rGO and WO_3_), which leads to a greater decrease in the resistance of the composite. In our case, these processes enhance the diffusion of charges at the WO_3_/rGO interface, but the role of nickel particles remains to be further clarified.

### Magnetic measurements

In order to investigate if nickel oxidation had occurred in the Ni@rGO composite during the heat treatment in the study of sensory properties (250 °C), the following model experiment was performed. The original Ni@rGO composite was annealed at a temperature of 250 °C for 2 h. In the following, its magnetic properties, namely the Curie temperature was determined from the temperature dependences of magnetization and magnetic susceptibility.

**Figure 7:**
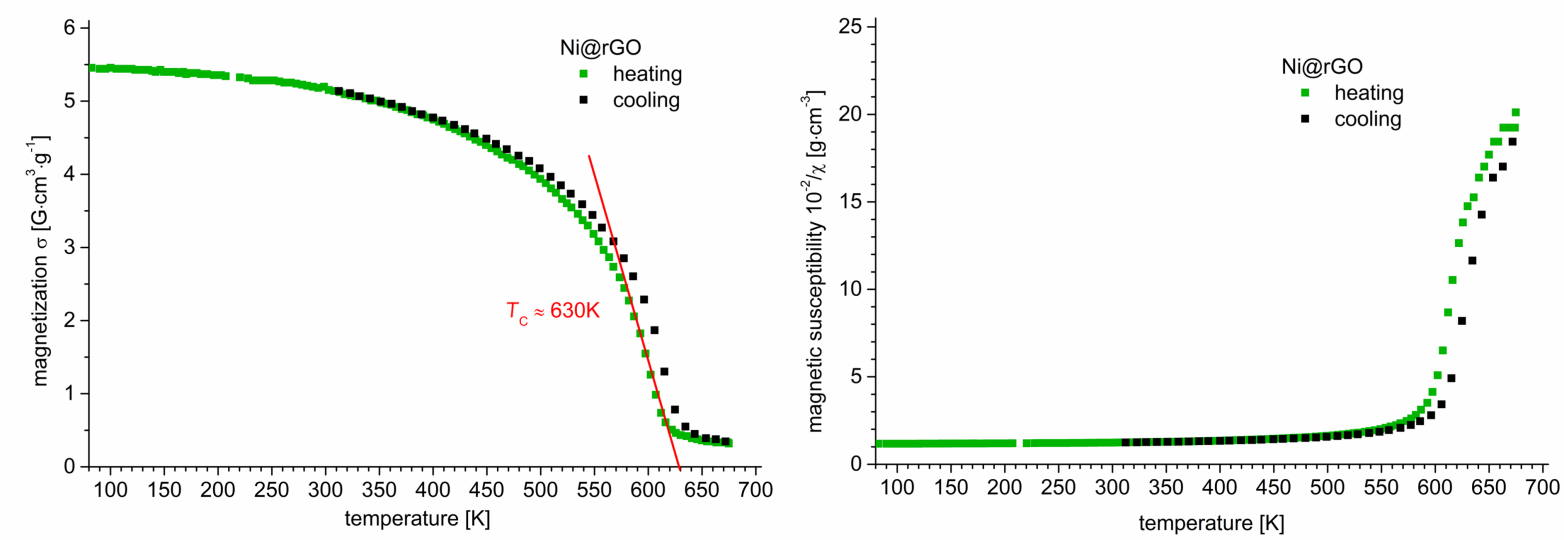
Left: magnetization as a function of temperature for the Ni@rGO composite in argon atmosphere. Right: magnetic susceptibility as a function of temperature for nickel in argon atmosphere.

The results of the magnetic analysis (Figure 7a) indicate that the magnetic phase in the Ni@rGO composite is pure nickel. The Curie temperature of the composite (*T*_c_ = 630 K, Figure 7, left) and the reference value for pure nickel [62] coincide (Figure S3, Supporting Information File 1). Moreover, the fraction of the magnetic phase in the Ni@rGO composite, as shown by magnetization measurements, is 7.8 wt %. The same amount of nickel is present in the original Ni@rGO composite without heat treatment.

The temperature dependence of the magnetization was determined during several heating–cooling cycles. It was noted that with an increase in the number of cycles, the Сurie temperature of the sample increased from 345 to 357 °C. Most likely, the increase in the Curie temperature is associated with an increase in the size of the nickel particles as a result of their sintering in agglomerates with an increase in the degree of crystallinity. The maximum temperature during cycling reached 450 °C. It can also be assumed that a sintering of nickel nanoparticles (approximately 25 nm in size), which are located in the partly formed agglomerates in the initial composite, is possible (Figure 2, right).

The increase in the Curie temperature with the size of nanoparticles is known [63–64]. It is explained by a decrease in the fraction of the nickel particle surface layer with a noncollinear spin configuration, which causes the formation of a surface spin canting due to thermal fluctuation of magnetic moments. Figure 7 shows the temperature dependence of the magnetization obtained in the course of the last measurement cycle at which, for nickel particles, ferromagnetic rather than superparamagnetic behavior is manifested. Hence, the results of measurements of the magnetic susceptibility and magnetization indicate the presence of only a metallic nickel phase in the Ni@rGO composite after heat treatment in air.

## Сonclusion

Ni@rGO nanocomposites were found to be promising materials that enable the preparation of WO_3_ gas and vapor sensor elements with improved sensory response. The addition of a very small amount of Ni@rGO (0.35 wt %) to WO_3_ increases the gas response regarding NO_2_ traces in air significantly compared to the WO_3_ element without the addition of metal and graphene oxide. Low concentrations of acetone (3500 ppm) were better detected by the Ni@rGO/WO_3_ composite than the higher concentration of 35,000 ppm. For CO gas, the response time and the recovery time were *T*_res_ ≈ 7 min and *T*_rec_ ≈ 2 min, respectively. The facile preparation of nickel nanoparticles supported on reduced graphene oxide paves the way for their application as dopant in other metal oxide gas sensors.

## Experimental

Due to the sensitivity of the precursor substances towards moisture and oxidation, all experiments were carried out in a purified argon (grade 99.998 vol %) or nitrogen (grade 99.996 vol %) atmosphere by using standard Schlenk techniques. Samples were prepared and stored in a MBraun Glovebox. Solvents (acetonitrile, *n*-hexane, and methylene chloride) were dried by using a MBraun solvent purification system or distilled (1-methylimidazole and 1-chlorobutane) and stored over 4 Å molecular sieves in a nitrogen atmosphere. Final water contents measured by coulometric Karl Fischer titration (ECH/ANALYTIK JENA AQUA 40.00) did not exceed 10 ppm.

Bis(1,5-cyclooctadiene)nickel(0) (Ni(COD)_2_) was purchased from ABCR, stored at −4 °C and used without further purification. The ionic liquid [BMIm][NTf_2_] was synthesized according to literature by reacting 1-methylimidazole with 1-chlorobutane to yield first [BMIm][Cl], which was further reacted with LiNTf_2_ to give [BMIm][NTf_2_] [65–66]. The IL was dried in a turbo molecular pump vacuum (10^−7^ mbar) at 80 °C for three days. Characterization was carried out by ^1^H and ^13^C NMR. Quantitative anion exchange and IL purity of 99.9% was assessed by ion chromatography (Dionex ICS-1100, with IonPac^®^ AS22, 4 × 250 mm column). The water content, measured by coulometric Karl Fischer titration, was below 10 ppm. rGO was synthesized in a two-step oxidation and thermal reduction process using natural graphite (type KFL 99.5 from AMG Mining AG, former Kropfmühl AG, Passau, Germany) as starting material. Graphite was oxidized according to [67]. Reduction of the graphite oxide was performed at 400 °C. Before using rGO in the nanoparticle synthesis, it was dried at 100 °C using a turbo molecular pump at 5 × 10^−7^ mbar for several days.

### Preparation of Ni@rGO in ionic liquid

Nickel nanoparticles on rGO (Ni@rGO) were prepared in septum-sealed 10 mL microwave vessels (CEM GmbH, Germany) in a CEM Discover microwave under argon atmosphere. Ni(COD)_2_ (49.2 mg, 0.178 mmol) and rGO (10 mg) were suspended for 2 h in the dried and deoxygenated IL (2 g [BMIm][NTf_2_]) before microwave decomposition (230 °C, 10 min, 50 W) to obtain a dispersion of 0.5 wt % of Ni nanoparticles on rGO in ionic liquid.

### Preparation of WO_3_ nanopowder

Tungsten oxide nanopowder was prepared according to [52] using the sol–gel method. A 1.23 mol/L aqueous solution of sodium tungstate dihydrate (Na_2_WO_3_⋅2H_2_O) was added into a 12 mol/L aqueous solution of nitric acid under constant rapid stirring. The prepared sol of tungstic acid was washed in distilled water using multiple centrifugation steps. After drying until the xerogel was formed, the WO_3_ nanopowder was calcined at 600 °C for 2 h.

### Ni@rGO mixing of WO_3_ xerogel

The Ni@rGO admixing of WO_3_ samples was done by preparing a physical mixture of the WO_3_ xerogel and Ni@rGO. At a pressure of 150 kPa, tablets were pressed from the powder (diameter 10 mm, thickness 2.5 mm, weight 0.75 g), which were sintered in air at 450 °C (4 h).

### Characterization

**Powder X-ray diffraction, P-XRD** data were measured at ambient temperature on a Bruker D2 Phaser using a flat sample holder and Cu Kα radiation (λ = 1.54182 Å, 35 kV). Samples had been precipitated with acetonitrile from the nanoparticle/ionic liquid dispersion and washed several times with acetonitrile. P-XRD patterns were recorded for 1 h (2θ = 5–100°).

**Atomic absorption spectroscopy, AAS** for metal analysis was performed on a PerkinElmer PinAAcle 900T, equipped with a flame furnace. Flame-AAS with an air/acetylene flame was used for the determination of the nickel content. Samples were digested in hot aqua regia two times (30 mL). The residues were re-dissolved in aqua regia, filtered and brought with water to a total volume of 10 mL. For the nickel measurements the samples were diluted 1:100.

**Transmission electron microscopy, TEM** was performed with a FEI Tecnai G2 F20 electron microscope [68] operated at 200 kV accelerating voltage or FEI Titan 80-300 TEM operated at 300 kV accelerating voltage [69]. Conventional TEM images were recorded with a Gatan UltraScan 1000P detector. TEM samples were prepared by drop casting the with acetonitrile diluted material on 200 µm carbon-coated copper grids, followed by washing the grid several times with acetonitrile to remove the excess ionic liquid. The size distribution was determined manually or with the aid of the Gatan Digital Micrograph software from at least 50 individual particles.

**Gas sensing properties** of the sensor elements were characterized using a custom-designed flow-type sensing measurement system inside a corundum chamber with precisely controlled temperature and atmosphere. Calibrated according to STB ISO 9001-2009, a CO/N_2_ mixture was received from Joint Stock Company "Kryon”. Liquid acetone was classified as “chemically pure” (purissimum). The NO_2_ gas was obtained by dissolving Cu in nitric acid (“chemically pure” grade). The CO/air mixture was measured in flow mode. NO_2_ and acetone were measured in a static mode. Here, a given amount of gas was introduced into a sealed chamber with a volume of 120 cm^3^. Electrical resistance of samples WO_3_, rGO/WO_3_, 0.35 wt % Ni@rGO/WO_3_ in the range of 20–240 °С was measured by the two-probe method in a corundum cell using an Agilent 34401 digital multimeter. The cell was placed in a tube furnace with a temperature regulator. To enhance electrical conductivity and to improve contact Ag electrodes were deposited on parallel sides of the pellets. The measurement procedure was carried out for NO_2_ and acetone in a stationary regime in a precisely controlled atmosphere (10 ppm NO_2_ and 3500 ppm acetone in air) according to the method proposed in [70]. The sensing element was placed into a preheated and thermostabilized chamber. Then, calibrated testing gas mixtures were injected into the chamber and measurements were carried out at the indicated temperature. Sensitivity to CO was measured in a dynamic regime using the CO/N_2_ gas mixture in flow mode. The CO/N_2_ mixture was fed at a rate of 2 L/h for aperiod of 10 min.

We note that it was not possible to set a repeated exposure to analyte gas and *T*_rec_ in a stationary mode for technical reasons. In the stationary regime, the flanges of the measuring cell have rubber plugs through which the required amount of analyte is injected from a microdispenser with a needle. To determine parameters such as *T*_rec_ and *T*_res_ in this stationary regime the operation of purging the cell with air would be necessary. For this, the cell flanges need to be replaced with other flanges. The temperature at which the measurements were carried out in the stationary mode excludes quick manipulations of these flanges. Even at room temperature, it takes 1 to 2 min to partially disassemble the chamber and replace the flanges with plugs with flanges with air purge pipes. Therefore, it was practically impossible to measure the relaxation time in a heated cell. Even at room temperature, at best, the measurement procedure will be incorrect, since the possible recovery time is comparable to the time of manipulations with the cell components.

**Magnetic measurements** were carried out by using the ponderomotive method with automatized installation for measuring magnetic characteristics and for the determination of magnetic impurities in substances by nondestructive testing with a precision to 0.01%. The measurement error for the specific magnetization of the measured samples is equal to ±0.005 A·m^2^/kg, for the magnetic susceptibility of samples with known mass it is equal to ±1 × 10^–11^ m^3^/kg.

## Supporting Information

File 1 Comparison with other gas sensors as well as the sensor response figures for CO and acetone.
