# Supplementary material for: Nickel nanoparticle-decorated reduced graphene oxide/WO3 nanocomposite – a promising candidate for gas sensing
Source: Beilstein J Nanotechnol. 2021 Apr 15;12:343–53. doi: 10.3762/bjnano.12.28 (PMC8056068; doi:10.3762/bjnano.12.28)
Supplement: File 1 — Comparison with other gas sensors as well as the sensor response figures for CO and acetone. [file Beilstein_J_Nanotechnol-12-343-s001.pdf]

BEILSTEIN JOURNAL OF NANOTECHNOLOGY

Supporting Information

for

Nickel nanoparticle-decorated reduced graphene oxide/WO_3 nanocomposite – a promising candidate for gas sensing

Ilka Simon, Alexandr Savitsky, Rolf Mülhaupt, Vladimir Pankov and Christoph Janiak

Beilstein J. Nanotechnol. **2021,** *12,* 343–353. doi:10.3762/bjnano.12.28

Comparison with other gas sensors as well as the sensor response figures for CO and acetone

License and Terms: This is a supporting information file under the terms of the Creative Commons Attribution License (https://creativecommons.org/licenses/by/4.0). Please note that the reuse, redistribution and reproduction in particular requires that the author(s) and source are credited and that individual graphics may be subject to special legal provisions.
The license is subject to the *Beilstein Journal of Nanotechnology* terms and conditions: (https://www.beilstein-journals.org/bjnano/terms)

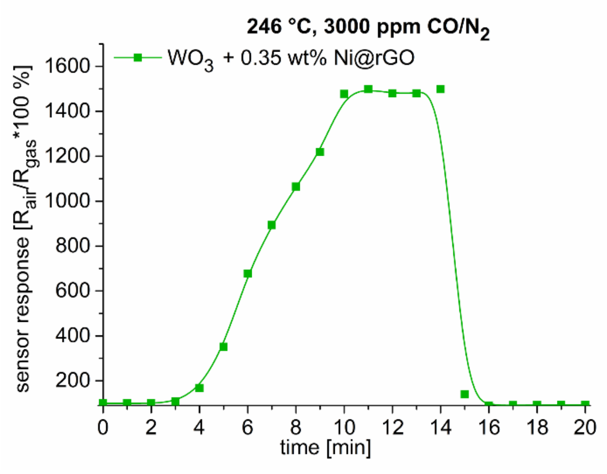

Figure S1: Time dependence of the sensor response of the 0.35 wt % Ni@rGO/WO_3 (green) samples under exposure to a CO vapor in nitrogen gas mixture at 3000 ppm. Response time: 0.35 wt % Ni@rGO/WO_3 (ca. 7 min.) Recovery time: 0.35 wt % Ni@rGO/WO_3 (ca. 2 min.)

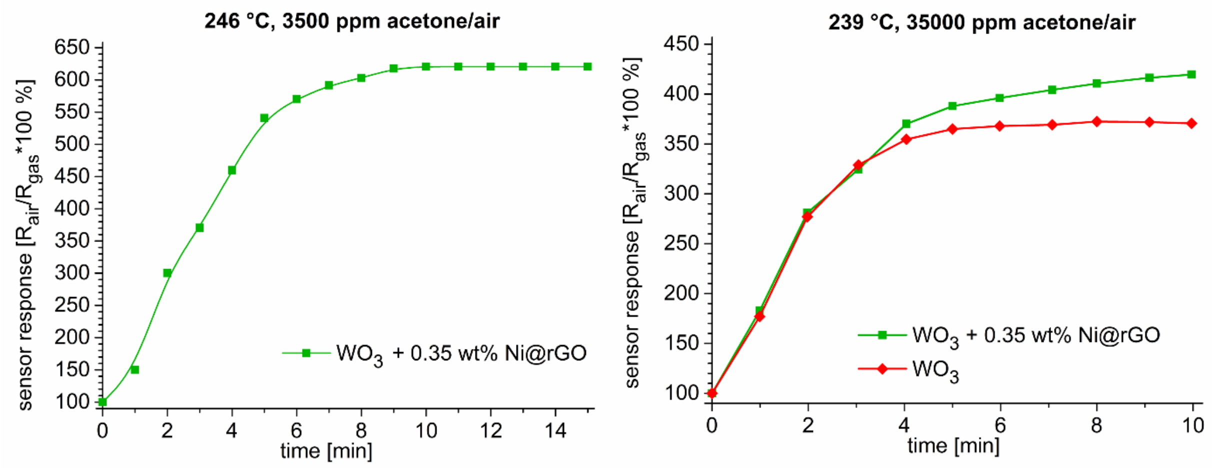

Figure S2: Left: time dependence of the sensor resistance values of the 0.35 wt % Ni@rGO/WO_3 samples (green) under exposure to an acetone vapor in air mixture at 3500 ppm. Right: time dependence of the sensor resistance values of the 0.35 wt % Ni@rGO/WO_3 (green) and WO_3 (red) samples under exposure to an acetone vapor in air mixture at 35000 ppm.

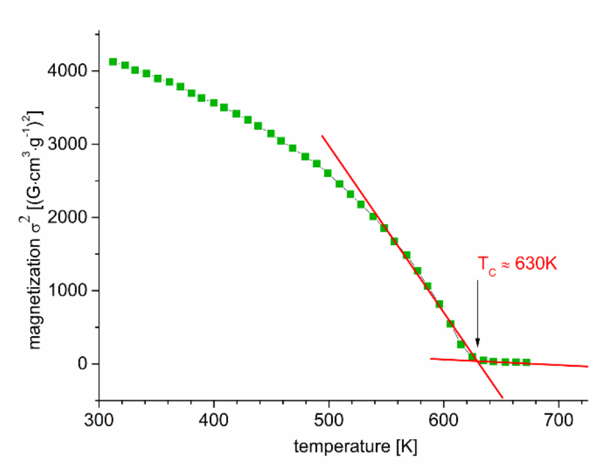

Figure S3: Square of the magnetization as a function of temperature for the reference sample of ferromagnetic nickel powder. Curie temperature T_c = 630 K.

Sensing materials – examples form literature

Table S1: A summary of graphene-based gas sensor characteristics for NO_2 gas.

Sensing Material	T_{res} (s)	Detection Limit	T_{rec} (s)	Ref.
GR	3000	4 / 100 ppb	3000	[1]
Ozone-treated GR	900	1.3 ppb	1800	[2]
rGO/hydrasine + WO_3	25	5 ppm (250 °C)	200	[3]
Multilayred GR	1800	6 / 1 ppm	–	[4]
rGO + NiO	125	200% / 1 ppm (200 °C)	250	[5]
FGO/$FeCl_3$+α-Fe_2O_3	80	180 ppb	44	[6]
rGO/hydrasine + ZnO	165	25.6 / 5 ppm (RT)	499	[7]
rGO+SnO_2 aerogel	190	50 ppm	224	[8]
SnO_2/S-rGO	40	26.3 / 20 ppm (RT)	357	[8]
rGO/$NaBH_4$	420	11.5% / 5 ppm	1680	[9]
rGO+SnO_2	75	3.3 / 5 ppm (50 °C)	300	[10]
rGO/WO_3	540	769% / 5 ppm (RT)	1080	[11]
rGO/In_2O_3	240	8.2 / 30 ppm	1440	[12]
WO_3/GR	–	202% / 20 ppm (300 °C)	–	[13]
MWCNTs/WO_3	10.5	77 / 5 ppm (RT)	20	[14]
WO_3/S-rGO	6	149% / 20 ppm (RT)	56	[15]
WO_3/rGO	250	40.8 / 56 ppm (RT)	–	[16]
WO_3/GR	0.42–3.3	96 / 1 ppm	0.42–3.3	[3]
NiO/SnO_2/rGO	220	62.27 / 60 ppm (RT)	835	[17]
(InOx/SnO_2)+rGO	400	22 / 100 ppm (RT)	–	[18]
FeOx/WO_3/rGO	1500	5.9 / 3 ppm (RT)	7200	[19]
Ag/rGO/SnO_2	49	2.17 / 5 ppm (RT)	339	[20]
0.35 wt % Ni/rGO/WO_3	540	295% / 10 ppm (240 °C)	–	this work
1.00 wt % Ni/rGO/WO_3	540	192% / 10 ppm (240 °C)	–	this work

Table S2: A summary of graphene-based gas sensor characteristics for acetone gas.

Sensing Material	T_{res} (s)	Detection Limit	T_{rec} (s)	Ref.
rGO/SnO_2 NFs	<198	100 ppb (350 °C)	<114	[21]
rGO/$ZnFe_2O_4$	4	10 ppm (275 °C)	18	[22]
Co_3O_4 NFs + Ir NPs + GO	–	1.18 / 120 ppb (300 °C)	–	[23]
SnO_2/rGO	198	1000 / 5 ppm	114	[21]
SnO_2/GO		200% / 200 ppm		[24]
CuO/ZnO/rGO		9.4 / 10 ppm		[25]
WO_3/Pt/rGO	14.1	12.2 / 10 ppm (200 °C)	16.8	[26]
NiO/SnO_2/GR	5.4	2.17 /200 ppm (150-350 °C)	150	[27]
0.35 wt % Ni/rGO/WO_3	660	620% / 3500 ppm (246 °C)	–	this work
0.35 wt % Ni/rGO/WO_3	660	420% / 35000 ppm (239 °C)	–	this work

Table S3: A summary of graphene-based gas sensor characteristics for CO gas.

Sensing Material	T_{res} (s)	Detection Limit	T_{rec} (s)	Ref.
CuO+rGO	70	2.56% / 1 ppm	160	[28]
0.35 wt % Ni/rGO/WO_3	420	1480% / 3000 ppm (in N_2)	120	this work

rGO – reduced graphene oxide, GO – Graphene oxide, GR – graphene; NPs – nanoparticles, NFs – nanofibers, DL – detection limit or response value at minimum measured concentration; T_{res} – period of time from gas sensor contact with gas to be detected to variation of resistance reach to 90% of R_a/R_g; T_{rec} – period of time from gas sensor away from gas to be detected to variation of resistance reach to 90% of R_a/R_g; RT = room temperature

References

[1] Yavari, F.; Castillo, E.; Gullapalli, H.; Ajayan, P. M.; Koratkar, N. *Appl. Phys. Lett.* **2012**, *100*, 203120. doi:10.1063/1.4720074

[2] Chung, M. G.; Kim, D. H.; Lee, H. M.; Kim, T.; Choi, J. H.; Seo, D. K.; Yoo, J.-B.; Hong, S.-H.; Kang, T. J.; Kim, Y. H. *Sens. Actuators, B* **2012**, *166-167*, 172–176. doi:10.1016/j.snb.2012.02.036

[3] Srivastava, S.; Jain, K.; Singh, V. N.; Singh, S.; Vijayan, N.; Dilawar, N.; Gupta, G.; Senguttuvan, T. D. *Nanotechnology* **2012**, *23*, 205501–205507. doi:10.1088/0957-4484/23/20/205501

[4] Choi, H.; Jeong, H. Y.; Lee, D.-S.; Choi, C.-G.; Choi, S.-Y. *Carbon Lett.* **2013**, *14*, 186–189. doi:10.5714/Cl.2013.14.3.186

[5] Le Hoa, T.; Tien, H. N.; van Luan, H.; Chung, J. S.; Hur, S. H. *Sens. Actuators, B* **2013**, *185*, 701–705. doi:10.1016/j.snb.2013.05.050

[6] Dong, Y.; Zhang, X.; Cheng, X.; Xu, Y.; Gao, S.; Zhao, H.; Huo, L. *RSC Adv*. **2014**, *4*, 57493– 57500. doi:10.1039/C4ra10136g

[7] Liu, S.; Yu, B.; Zhang, H.; Fei, T.; Zhang, T. *Sens. Actuators, B* **2014**, *202*, 272–278. doi:10.1016/j.snb.2014.05.086

[8] Liu, X.; Cui, J.; Sun, J.; Zhang, X. *RSC Adv.* **2014**, *4*, 22601–22605. doi:10.1039/C4RA02453B

[9] Su, P.-G.; Shieh, H.-C. *Sens. Actuators, B* **2014**, *190*, 865–872. doi:10.1016/j.snb.2013.09.078

[10] Zhang, H.; Feng, J.; Fei, T.; Liu, S.; Zhang, T. *Sens. Actuators, B* **2014**, *190*, 472–478. doi:10.1016/j.snb.2013.08.067

[11] Su, P.-G.; Peng, S.-L. *Talanta* **2015**, *132*, 398–405. doi:10.1016/j.talanta.2014.09.034.

[12] Gu, F.; Nie, R.; Han, D.; Wang, Z. *Sens. Actuators, B* **2015**, *219*, 94–99. doi:10.1016/j.snb.2015.04.119

[13] An, X.; Yu, J. C.; Wang, Y.; Hu, Y.; Yu, X.; Zhang, G. *J. Mater. Chem.* **2012,** *22*, 8525–8531. doi:10.1039/c2jm16709c

[14] Su, P.-G.; Pan, T.-T. *Mater. Chem. Phys.* **2011**, *125*, 351–357. doi:10.1016/j.matchemphys.2010.11.001

[15] Wang, T.; Hao, J.; Zheng, S.; Sun, Q.; Di Zhang; Wang, Y. *Nano Res.* **2018**, *11*, 791–803. doi:10.1007/s12274-017-1688-y

[16] Jie, X.; Zeng, D.; Zhang, J.; Xu, K.; Wu, J.; Zhu, B.; Xie, C. *Sens. Actuators, B* **2015**, *220*, 201–209. doi:10.1016/j.snb.2015.05.047

[17] Zhang, J.; Wu, J.; Wang, X.; Zeng, D.; Xie, C. *Sens. Actuators, B* **2017**, *243*, 1010–1019. doi:10.1016/j.snb.2016.12.062

[18] Cui, S.; Wen, Z.; Mattson, E. C.; Mao, S.; Chang, J.; Weinert, M.; Hirschmugl, C. J.; Gajdardziska-Josifovska, M.; Chen, J. *J. Mater. Chem. A* **2013**, *1*, 4462–4467. doi:10.1039/c3ta01673k

[19] Piloto, C.; Shafiei, M.; Khan, H.; Gupta, B.; Tesfamichael, T.; Motta, N. *Appl. Surf. Sci.* **2018**, *434*, 126–133. doi:10.1016/j.apsusc.2017.10.152

[20] Wang, Z.; Zhang, Y.; Liu, S.; Zhang, T. *Sens. Actuators, B* **2016**, *222*, 893–903. doi:10.1016/j.snb.2015.09.027

[21] Choi, S.-J.; Jang, B.-H.; Lee, S.-J.; Min, B. K.; Rothschild, A.; Kim, I.-D. *ACS Appl. Mater. Interfaces* **2014**, *6*, 2588–2597. doi:10.1021/am405088q

[22] Liu, F.; Chu, X.; Dong, Y.; Zhang, W.; Sun, W.; Shen, L. *Sens. Actuators, B* **2013**, *188*, 469–474. doi:10.1016/j.snb.2013.06.065

[23] Choi, S.-J.; Ryu, W.-H.; Kim, S.-J.; Cho, H.-J.; Kim, I.-D. *J. Mater. Chem. B* **2014**, *2*, 7160–7167. doi:10.1039/c4tb00767k

[24] Han, M.; Liu, W.; Qu, Y.; Du, L.; Wei, H. *J. Mater. Sci.: Mater. Electron.* **2017**, *28*, 16973–16980. doi:10.1007/s10854-017-7619-6

[25] Wang, C.; Zhu, J.; Liang, S.; Bi, H.; Han, Q.; Liu, X.; Wang, X. *J. Mater. Chem. A* **2014**, *2*, 18635–18643. doi:10.1039/C4TA03931A

[26] Chen, L.; Huang, L.; Lin, Y.; Sai, L.; Chang, Q.; Shi, W.; Chen, Q. *Sens. Actuators, B* **2018**, *255*, 1482–1490. doi:10.1016/j.snb.2017.08.158

[27] Singkammo, S.; Wisitsoraat, A.; Sriprachuabwong, C.; Tuantranont, A.; Phanichphant, S.; Liewhiran, C. *ACS Appl. Mater. Interfaces* **2015**, *7*, 3077–3092. doi:10.1021/acsami.5b00161

[28] Zhang, D.; Jiang, C.; Liu, J.; Cao, Y. *Sens. Actuators, B* **2017**, *247*, 875–882. doi:10.1016/J.SNB.2017.03.108
